# Quantitative nanoscale MRI with a wide field of view

**DOI:** 10.1038/s41598-019-47084-w

**Published:** 2019-08-21

**Authors:** F. Ziem, M. Garsi, H. Fedder, J. Wrachtrup

**Affiliations:** 1 3rd Physical Institute, University of Stuttgart, Stuttgart, Germany; 2 Institute for Quantum Science and Technology, IQst, Baden-Württemberg, Germany; 3 Center for Applied Quantum Technology, Stuttgart, Germany; 4 Swabian Instruments GmbH, Stammheimer Str. 41, 70435 Stuttgart, Germany

**Keywords:** Solid-state NMR, Quantum metrology, Wide-field fluorescence microscopy, Super-resolution microscopy

## Abstract

Novel magnetic sensing modalities using quantum sensors or nanoscale probes have drastically improved the sensitivity and hence spatial resolution of nuclear magnetic resonance imaging (MRI) down to the nanoscale. Recent demonstrations of nuclear magnetic resonance (NMR) with paramagnetic colour centres include single molecule sensitivity, and sub-part-per-million spectral resolution. Mostly, these results have been obtained using well-characterised single sensors, which only permit extended imaging by scanning-probe microscopy. Here, we enhance multiplexed MRI with a thin layer of ensemble spin sensors in an inhomogeneous control field by optimal control spin manipulation to improve ensemble sensitivity and field of view (FOV). We demonstrate MRI of fluorine in patterned thin films only 1.2 nm in thickness, corresponding to a net moment of 120 nuclear spins per sensor spin. With the aid of the NMR signal, we reconstruct the nanoscale depth distribution of the sensor spins within the substrate. In addition, we exploit inhomogeneous ensemble control to squeeze the point spread function of the imager to about 100 nm and show that localisation of a point-like NMR signal within 40 nm is feasible. These results pave the way to quantitive NMR ensemble sensing and magnetic resonance microscopy with a resolution of few ten nanometers.

## Introduction

Nitrogen-vacancy (NV) colour centres in diamond allow for optical polarisation and readout of their electron spin state, which enables electron paramagnetic resonance (EPR) using single spins. Direct control over the NV spin is exerted by microwave (MW) irradiation, and various experimental schemes have been employed to use individual or ensemble NVs to probe magnetic^1^ and electric fields^2^, as well as pressure^3^ and temperature^4^. Remarkably, these capabilities persist over a wide range of conditions, including ambient conditions and e.g. cellular environments, providing magnetic sensing and imaging capabilities in lab-on-a-chip devices^5,6^. The small sample-to-sensor distance obtained with quantum sensors and nanoscale probes has enabled NMR on zeptoliter volumes^7–10^ and single molecules^11,12^. Extensions to the underlying quantum sensing protocol have yielded chemical resolution^13–16^.

**Figure 1:**
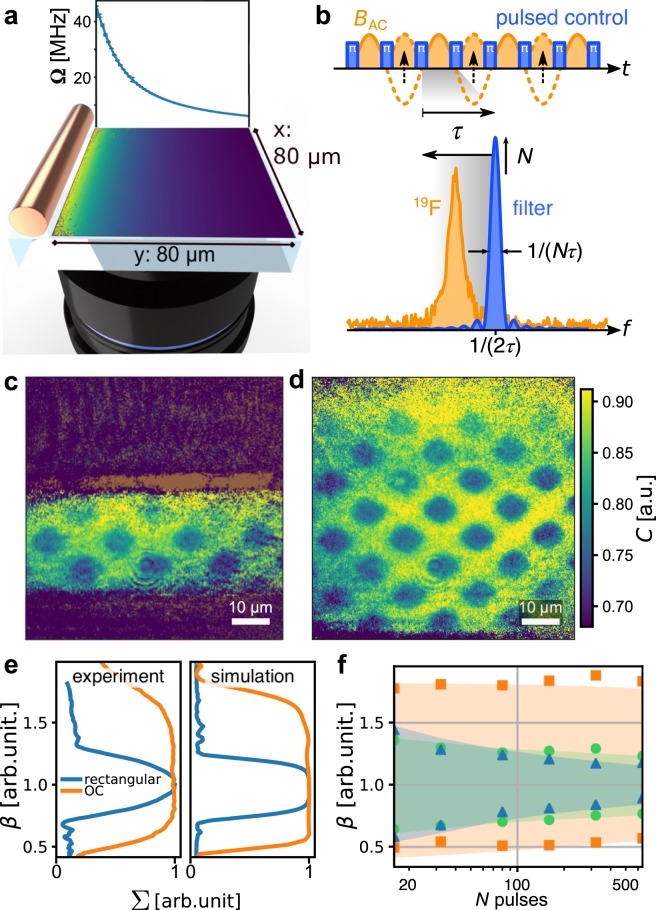
Experimental setting. (**a**) A 20 μm thick wire for applying microwave control fields was placed next to the field of view of the magnetic imager. The *y*^−1^ decay of the field amplitude away from the wire resulted in a range of NV Rabi frequencies across the field of view. (**b**) DD lock-in sensing utilises a train of *N π*-pulses to lock into a signal with period 1/*f* = 2*τ*. This creates a filter which scales in width as 1/(*Nτ*) and in height as *N*. (**c**,**d**) Lock-in MRI of ^19^F nuclei in patterned CaF_2_ on the diamond surface (dark blue squares in the image) using rectangular and optimal control pulses, respectively. The MW wire was located parallel to the horizontal edge at the bottom, outside of the field of view. While the gradient was steep at the bottom of the image, it was moderate at the top, leading to a gradual loss of contrast at the top of the effective range, where the fidelity of the decoupling decayed. (**e**) Experimental and simulated decoupling efficacy ∑ across the gradient (averaged along *x*) for *N* = 320 pulses. (**f**) Experimental width (FWHM of profiles as in e) of the range within the gradient covered by XY16-*N* using rectangular (triangles), Knill (circles) and optimal control pulses (squares). Shaded areas correspond to results of the simulations.

**Figure 2:**
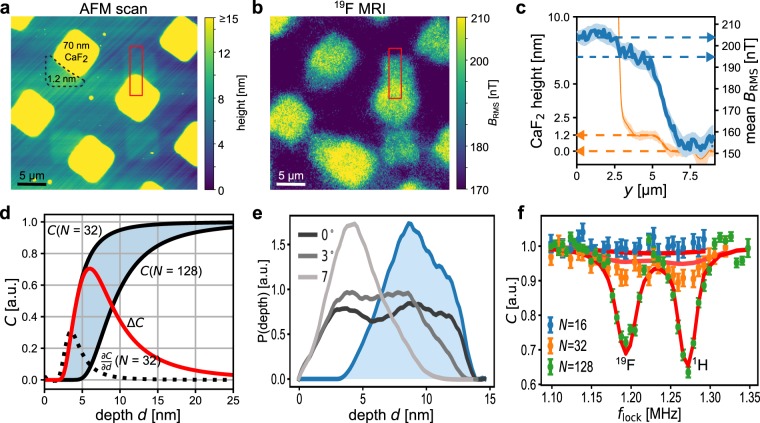
Nuclear magnetic resonance imaging. The diamond surface was patterned with CaF_2_ islands. (**a**) AFM scan of the diamond surface. (**b**) Approximate effective magnetic field amplitude *B*_rms_ measured with XY16-256. (**c**) Profiles of the surface (orange, AFM data) and ^19^F *B*_rms_ (blue, magnetometry data) along a transition from 70 nm CaF_2_ to 1.2 nm CaF_2_ to the bare diamond at *y* = 3 and 5.5 μm, respectively. (**d**) Calculated on-resonance lock-in contrast *C*(*d*) to reconstruct the distribution of NV depths in the ensemble. The coherence loss due to the nuclear phase noise depends on NV depth and the duration of the lock-in (given by *N*). Single measurements have a limited dynamic range (derivative ∂*C*/∂*d*, dotted black line), while the integral over pairwise differences (shaded blue) contains information about intermediate depths (difference in contribution to ensemble contrast, solid red line). (**e**) Recovered distribution of NV depths (blue, filled), based on CTRIM simulations of N^+^ implantation for 2.5 keV at angles 0°, 3°, and 7° against the surface normal (grey to black lines, respectively), as well as a depletion zone. (**f**) Ensemble averaged data for NVs below 70 nm CaF_2_ and fit using the recovered distribution of depths.

Nanoscale NMR with spin sensors is mostly realised by quantum phase acquisition in interferometric dynamical decoupling (DD) sequences^7^, which consist of a train of *N* MW pulses, applied at rate *τ*^−1^, and increase sensitivity to field fluctuations within a narrowed frequency window around a lock-in frequency *f*_lock_ = (2*τ*)^−1^ (Fig. 1b). The NMR signal arises from statistical polarisation of nuclear spins as an oscillating magnetic field *B*_AC_(*t*) at the nuclear Larmor frequency *f*_n_, which for *f*_lock_ ≈ *f*_n_ is observed as decoherence of the NV sensor when averaged over uncorrelated measurements. The corresponding spectral features can be described as relative contrast $1\ge C=\exp (-\frac{2}{\pi }\,{B}_{{\rm{rms}}}^{2}(d)\,K(N\tau ))\ge 0,$ see Fig. 2f. The root mean square amplitude *B*_rms_ ∝ *d*^−3^ of *B*_AC_ scales strongly with distance *d* of the NV sensor from the sample. The line shape *K*(*Nτ*) is the convolution of the spectral density of the nuclear magnetic signal with the DD filter. A detailed description has been been provided by Pham *et al*.^17^

Nanoscale MRI has been demonstrated by scanning a single NV centre across a sample specimen^18,19^, but comes with the inherent drawback of slow image acquisition speeds due to long interrogation times at each pixel. This limitation can be overcome in a wide field imaging mode to record the fluorescence response of a large ensemble of NV centres in parallel, which has been demonstrated in several applications of magnetic imaging^5,6,20–22^, including micron-scale MRI^23^. To unleash the full power of parallelised sensing, it is key to reliably control the ensemble of NVs over its entire volume despite inhomogeneities of the MW drive strength and magnetic resonance frequencies. These are caused by (i) the microwave structures, especially in tightly integrated devices, and (ii) the inhomogeneity of the static magnetic field, hyperfine coupling and orientations of the spin quantisation axis. We focused on the first point and manipulated a homogeneously implanted NV ensemble with MW pulses applied through a thin wire with a diameter of 20 μm as a test bed for gradients in the driving field encountered in integrated systems or volume sensors, see Fig. 1a. We mapped the NV Rabi frequency Ω to position and observed the gradient along the *y*-coordinate perpendicular to the wire, but little variance along *x*, parallel to the wire. This setup enabled imaging, or averaging of equivalent pixels, e.g. to obtain the Rabi frequency Ω at different distances from the wire, extending from less than 10 MHz to above 40 MHz (Fig. 1a).

Approaches to improve the robustness of spin control include specific microwave structures^24,25^ or improved phase cycles in DD sequences^26,27^. In contrast, we aimed to obtain building blocks for arbitrary control sequences and experimental settings by improving the robustness of the individual pulses. Robust pulses include composite pulses^28^ or pulses derived using optimal control theory^10,29,30^. We employed the GRAPE algorithm^29,31^ to engineer optimal control (OC) *π* and *π*/2 rotations specifically for our spin ensemble (see Methods section).

## Results

In Fig. 1c,d we demonstrate the shortcomings of regular pulsed control within ensemble settings, by MRI using rectangular and OC *π*-pulses, respectively, applied in XY16 sequences^26^. We imaged ^19^F in a structure of CaF_2_ patches with a thickness of 70 nm, visible as dark rectangles with reduced relative contrast *C*. We chose a reference Rabi frequency Ω_0_ = 20 MHz, roughly the median value of the range of Rabi frequencies observed within the MW gradient. We defined a relative strength of the driving field *β* = Ω/Ω_0_, which continuously decreased as the distance from the wire increased. We applied dynamical decoupling with rectangular *π*-pulses of duration 1/(2Ω_0_) = 25 ns which resulted in successful decoupling and imaging within a range *β* ≈ 0.8 to 1.2, shown in Fig. 1c. Clearly, the OC pulses extended the control over almost the complete ensemble within *β* ≈ 0.5 to 1.7, Fig. 1d. On the other hand, at a duration of 122 ns, the OC pulses reduced the per-sensor sensitivity of NVs which were already decoupled well with rectangular pulses. Assuming that there is no accumulation of phase during each *π*-pulse of duration *t*_*π*_, each free evolution segment in the DD sequence reduces to *τ*′ = *τ* − *t*_*π*_, since the rate of pulses is unchanged to stay in resonance with the nuclear signal. Compared to an instantaneous *π*-pulse, the acquired phase with a pulse of finite duration reduces to a fraction $\kappa =\frac{\tau ^{\prime} }{\tau }({\rm{sinc}}(\frac{\tau ^{\prime} }{2\tau })/\mathrm{sinc}(\frac{1}{2}))$, where ${\rm{sinc}}(x)\equiv \frac{\sin (\pi x)}{\pi x}$. As a result, the minimum detectable *B*_rms_ in noise spectroscopy increases as 1/*κ* for a given sensor within the ensemble. Assuming shot noise, the longer OC pulses need to increase the fraction of the ensemble which contributes to the signal by 1/*κ*^4^ to still achive a net gain in sensitivity^32^ (Supplementary Information for details). In our case, the OC pulses improved the net sensitivity, since *κ*_rect_ ≈ 1 for the rectangular pulse, *κ*_OC_ ≈ 0.84 for the OC pulse, and we observed an increase in FOV by a factor of about $2.2 > 1/{\kappa }_{{\rm{OC}}}^{4}\approx 2$. In applications requiring time resolved imaging, at least two measurements would have been necessary with rectangular pulses to image across the whole FOV, resulting in a critical reduction of the frame rate.

Complementary to the MRI result, we measured full XY16 *T*_2_ decoherence traces over a wider range of interpulse spacings *τ* and a range of pulse numbers *N* on the same diamond sample. In those areas of the FOV, where decoupling worked well, the decay of the spin state contrast over *τ* was roughly described by exponential decays *A*exp(−*τ*/*T*_2_), with amplitude *A* at *τ* = 0. We numerically integrated the *T*_2_ data to obtain an index ${\rm{\Sigma }}\approx {\int }_{0}^{\infty }\,A\exp (\,-\,\tau /{T}_{2}){\rm{d}}\tau =A{T}_{2}$, such that higher values reflect better sensitivity (Supplementary Information for details). Figure 1e shows the normalised values of ∑ for *N* = 320 pulses across the gradient, as obtained from experiment and numerical simulations. From the good agreement between experiment and simulations, which included only *β* and ^15^N hyperfine interaction, we conclude that the range of effective decoupling was dominated by pulse errors and not by relaxation due to the spin bath or additional hyperfine interaction. We also added the performance of the composite Knill pulse^26,33^. Its total duration of 125 ns is similar to that of the OC pulse, but the Knill pulse was effective only across a similar range of *β* as the rectangular pulses, albeit exhibiting more flat-top ∑ profiles (not shown). Figure 1f summarises the results by showing the full width at half maximum (FWHM) of the ∑ profiles for all measurements and corresponding simulations. For the rectangular pulses, the FWHM quickly narrowed down with increasing number of pulses and reached a value of Ω_0_ ± 15% for 640 pulses. The optimal control pulses performed equally well across all applied pulse numbers. *T*_2_ scaled similarly for all pulse types around *β* ≈ 1, with *T*_2_ = 9.4(6) × *N*^0.43(1)^ on average.

Using the characterized OC pulses, we take a closer look at the MRI results, see Fig. 2. We added a second pattern of CaF_2_ with a thickness of 1.20(5) nm, as confirmed by atomic force microscope (AFM) measurements, Fig. 2a. The MRI results, Fig. 2b, included a constant ^19^F signal across the whole diamond which had been absent on the untreated sample, but which remained after initial NMR measurements with a polytetrafluoroethylene (PTFE) coating on the sample (not shown here). Notably, the thin deposited islands are clearly visible against this background. In addition, a proton signal was observed, which is typically attributed to water adsorbed on the diamond surface.

In the following, we describe the reconstruction of the NV depth distribution based on the MRI measurements. Only with this knowledge, interpretation of the NMR contrast *C* in terms of the average *B*_rms_ became possible (see Fig. 2b,c). Within an ensemble measurement, the NV fluorescence, which encodes the NMR signal, is averaged over NVs at different depths below the diamond surface. To interpret the observed NMR contrast *C* quantitatively in terms of e.g. spin densities and average magnetic field strength, knowledge of the distribution of sensor depths is required. The depth distribution of implanted NVs strongly depends on the exact implantation parameters, see e.g. Figure 2e, but can be determined from the MRI data^17^. Here we demonstrate, how the method can be extended from the case of single to ensemble NVs. The mean contrast $\bar{C}$ in an ensemble measurement with *N* pulses is weighted by *P*(*d*), the distribution of depths: ${\bar{C}}_{N}={\int }_{0}^{\infty }\,P(d){C}_{N}(d){\rm{d}}d,$ where we emphasize the role of *N* by adding it as a subscript. Due to strong dependence of the NMR contrast on the NV depth, the NV density can be reconstructed by combining several NMR measurements with different *N*. For two different *N* = *A*, *B*, the change in the observed contrast is given by $\overline{{\rm{\Delta }}C}={\int }_{0}^{\infty }{P}_{d}(d)\,{\rm{\Delta }}C\,{\rm{d}}d={\int }_{0}^{\infty }{P}_{d}(d)[{C}_{A}(d)-{C}_{B}(d)]{\rm{d}}d,$ as illustrated in Fig. 2d. A set of measurements with different *N* then defines a set of equations from which *P*(*d*) can be inferred by fitting a model distribution or in a parameter-free approach. To reconstruct the distribution of NV depths of the 2.5 keV implantation used in our experiments, we combined MRI data with *N* = 16, 32, 128, 160, and 256. We based the reconstruction on a small set of CTRIM simulations of the nitrogen implantation^34^ to keep the number of free parameters low, but at the same time account for implantation effects like channeling of ions along certain crystal axes. We added an additional depletion zone for NVs close to the diamond surface, which are typically of low stability. The resulting distribution is shown in Fig. 2e, together with the CTRIM simulations for an implantation energy of 2.5 keV at implantation angles 0°, 3°, and 7°, which served as the basis for reconstruction. The implantation angle during NV creation was not known exactly and expected to deviate around 3° from zero, while the reconstruction suggests an angle close to 0°. The distribution had its mode at 8.6 nm, and a standard deviation of 2.2 nm, matching previous statistics gathered on single NV centres^17^. The spectral features calculated based on the reconstructed depth distributions, match the data points for *N* = 128 well, while for *N* = 32 the experimental contrast is stronger than reconstructed, see Fig. 2f. We conclude, that our reconstruction slightly underestimates the NV density for depths between about 3 to 6 nm since this region provides the contrast for *N* = 32, see Fig. 2d. In addition, at the deeper end of the distribution, it is clearly limited by the extend of the underlying CTRIM simulations. Yet, in summary, reasonable parameters were obtained in the reconstruction (see Methods).

**Figure 3:**
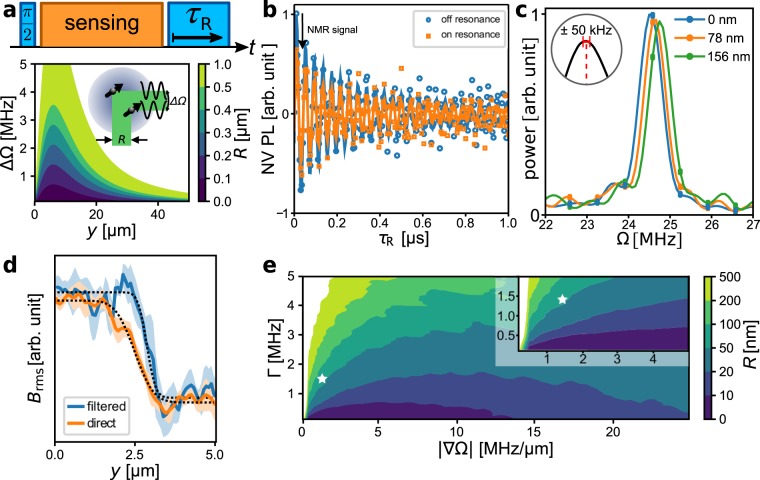
Superresolution in MW gradient. (**a**) A spin state projection pulse with variable duration *τ*_R_ results in an oscillating sensor response, modulated by the sensing result. Discrimination between spectral components with resolution ΔΩ translates to spatial resolution *R* at a given distance *y* from the wire, which is located at *y* = 0. (**b**) The amplitude of the Rabi readout signal is modulated by the ^19^F NMR signal. On resonance, a reduced contrast is obtained. Data are from a single camera pixel, solid lines are least-squares fits. (**c**) Periodograms (with interpolation) of the Rabi oscillations from adjacent pixels clearly reflect the shift of the Rabi frequency within the gradient. Note that upsampling of the frames reduced the effective pixel size, see main text. Inset: Typical uncertainty of the line centres according to the least-squares fits in the time domain. (**d**) Fitting the NMR signal after filtering the spectra recovers sharpened steps at CaF_2_ edges. (**e**) Simulated spatial resolution *R* for a range of gradient strengths |∇Ω| and intrinsic spectral linewidths Γ. Star marks experimental conditions. Inset: zoom.

Finally, we used the spatial encoding via the Rabi frequencies in the gradient to improve the spatial resolution of the MRI^35,36^. The width of the point spread function (PSF) of spin noise NMR itself is lower-bounded by the distance between NV and sample to *σ*_NMR_ ≈ 10 nm as shown by scanning-probe MRI^18,19^. Within the wide field setting, this value is dominated by the width of the optical PSF of the NV imager, *σ*_opt_ ≈ 165 nm. Several studies enhancing the optical localisation of NV centres using superresolution techniques like STED^37,38^ and STORM^39^ have been published, reaching localisation down to a few nanometers. Combining a large field of view with nanoscale resolution can be achieved by Fourier imaging, which uses spatial encoding via gradient fields^40,41^. Technically, one is often limited to gradients of a few Gauss/micron and nanoscale resolution thus requires phase accumulation over an extended time period. Here we use the driving field inhomogeneity itself as gradient source to improve the spatial resolution along the direction of the gradient. We modified the spin projection at the end of the NMR sequence and replaced the final *π*/2 pulse with a pulse of variable duration *τ*_R_, see Fig. 3a. As a result, we observed Rabi oscillations of the sensor, which were modulated in amplitude by the result of the sensing sequence, here by the ^19^F NMR signal, as shown in Fig. 3b. The power spectra of these oscillations, Fig. 3c, shifted notably between adjacent pixels in a gradient of |∇Ω| ≈ 1.5 MHz/μm (equivalent to 50 μT/μm). Note that we applied drift correction to the frames obtained with the imager, during which we upsampled them by a factor of two, reducing the effective pixel width by half (see Methods and Supplementary Information for details). The uncertainties of the least-squares fit to the Rabi oscillations in Fig. 3b where 50 kHz, a value which allows to localise a single NV along the direction of the gradient to within 30 nm, a factor of 5.6 below the diffraction limit. Taking into account the depth of our NVs of about 10 nm below the surface, we conclude that we can localise an NMR signal within 40 nm.

In the ensemble, the Rabi spectrum at each pixel contains the contributions from NVs within reach of the optical PSF, which may not be individually resolvable in case of high NV densities, but nevertheless we show that improved spatial resolution can be achieved. By band-filtering the spectra to a narrow range around the expected Rabi frequency Ω(*y*) in the gradient, contributions from other positions are largely rejected. The filtered signal corresponds to the NMR signal from a narrowed PSF. We filtered the spectra around the expected Rabi frequencies to a band given by gradient multiplied by the effective pixel size of about 78 nm obtained after upsampling. E.g. at |∇Ω| ≈ 1.5 MHz/μm the filter width was |∇Ω| × 0.078 μm = 0.12 MHz. By defining the bandwidth in this way, the spectrum was continuously sampled across the gradient and the signal from all NVs located within a pixel were included. We examined the step of the NMR signal at the edges of the 1.2 nm CaF_2_ islands in this way. From the AFM measurements, we found a width of the CaF_2_ edges of below 0.3 μm, including convolution with the AFM tip (see Methods). We compared the step widths in the NMR signal at six different CaF_2_ islands and found widths of 0.24(1) μm and 0.29(3) μm with and without Rabi readout, respectively. Figure 3d shows an example step. By simulation of the NMR signal due to the CaF_2_ step, we found that the likely step width of CaF_2_ is 0.21(2) μm (excluding the AFM tip), resulting in an effective width of the PSF of the Rabi readout *σ*_RR_ ≈ 80 to 120 nm, a factor of 1.7 below the diffraction limit. We attribute the difference compared to the factor 5.6 above to remaining overlap between the spectra of different NVs along the gradient. Simulations of the spatial mapping of a given spectral line suggest a resolution of about 70 nm for our experimental conditions, see Fig. 3e. The experimental value is slightly increased compared to this results, which we mainly attribute to remaining uncorrected drift of the experimental setup and spectral overlap of NVs along the gradient within a diffraction limited spot. In principle, reduction of the band used for filtering the spectra further reduces the spatial extend from which the filtered signal is drawn and improves resolution, but we did not observe any notable improvement in resolution upon doing so and expect to be mainly limited by the linewidths. Further improvements are thus expected from stronger gradients and improved engineering of shallow NV ensembles.

## Discussion

We have shown in this work that bolstering dynamical decoupling sequences with robust pulses enables control of spin ensembles even in adverse experimental conditions in terms of driving field inhomogeneity. Optimal control pulses can be tailored to a well characterised system and may give a decisive advantage in applications which e.g. set tight boundaries on the power of the applied pulses. Our chosen geometry, using a wide field approach, enabled us to characterise the performance of an optimal control pulse quickly. The gradient in MW amplitude could in the future be accompanied by a second gradient at right angle, e.g. of the static magnetic field, to map the performance of the pulses across a range of detunings in parallel. This hints at the potential application of wide field imagers for multiplexed probing of any quantity accessible to the NV (magnetic and electric fields, temperature, strain) across a large set of combinations of at least two controlled parameter gradients. We applied the optimised pulses in NMR microscopy and imaged thin films containing fluorine resulting in an effective signal of about 120 nuclei. From the experimental signal-to-noise ratio, we expect to be able to clearly image layers down to 0.5 nm in thickness, corresponding to about 80 nuclei, within four hours measurement time. We have also introduced a modified readout scheme, in which the result of the measurement sequence can be encoded into the amplitude and/or phase of a periodic NV response. We demonstrated improved spatial resolution within modest gradients, but further substantial improvements in resolution are expected in a stronger gradient, either closer to the MW structure or by an increase in MW power, as well as with longer intrinsic lifetimes of the Rabi oscillations. Simulations of ensemble Rabi readout NMR measurements in a gradient of 5 MHz/m resulted in a step width of 40 nm for MRI of an edge (Supplementary Information). The Rabi readout scheme may be incorporated into existing confocal and wide field superresolution techniques or as an ad-hoc way to utilise inhomogeneities which are already present in the system.

## Methods

### NV ensemble

The diamond sample was an ultrapure CVD grown monocrystal with natural abundance of ^13^C (element6) with a thickness of 20 μm. We created NV centres by implantation of ^15^N^+^ at a fluence of 10^12^ cm^−2^ and energy of 2.5 keV. The exact implantation angle against the surface normal was not known, but was expected to deviate around 3° from zero. Implantation was followed by annealing at 800 °C for 2 h. From the implantation parameters, we estimate a lateral NV density of about 20 μm^−2^. We measured an ensemble average *T*_2_ = 12(3) µs and obtained a sensitivity of $0.9(2)\,{\rm{\mu }}{\rm{T}}/(\sqrt{{\rm{Hz}}}\,{\rm{\mu }}{\rm{m}})$ within the central 600 μm^2^ of the field of view from the signal-to-noise ratio in XY16-160 measurements.

### Experimental setup and measurement

The experimental realisation of the optimal control pulses was achieved by IQ modulation of an MW source (Rhode&Schwarz SMBV100A) using an arbitrary waveform generator (AWG) (Tektronix AWG520). All pulses, including the standard rectangular pulses, were generated using this combination. The AWG was set to a clocking rate of one gigasample per second, the same time step used in the optimal control pulses. In parallel to pulse shaping, the AWG also controlled an MW switch (MiniCircuits) between MW source and amplifier to suppress pulse transients and spurious signals during free evolution times between the pulses. Finally, the generated MW signal was sent through a band pass filter (MiniCircuits), amplified (100S1G4, Amplifier Research) and applied to the wire. All MW components were operated well below their saturation powers. We determined the modulation bandwidth (200 MHz) and pulse shape after passing through the whole MW chain and found no limitations.

The microscope consisted of a 60× oil immersion objective with NA = 1.49 (Olympus), a 650 nm long-pass filter (Omega), a 300 mm tube lens and a Cascade II:512 CCD camera (512 × 512 pixels, Photometrics), resulting in a field of view of 80 μm × 80 μm and an effective pixel width of about 156 nm on the object side. Since the NVs were imaged through the diamond, the high refractive index of diamond effectively reduced the NA of the objective. Based on refraction of the edge ray just captured by the objective at the interface between diamond and immersion oil, we estimate an effective NA of 0.94, resulting in a standard deviation of *σ*_opt_ ≈ 0.22*λ*/NA ≈ 165 nm of the normal distribution approximating the point spread function. Here, *λ* ≈ 700 nm is at the centre of the detection band. A 532 nm laser (Coherent) and an acousto-optical modulator (Crystal Technology) were used for pulsed excitation of the NVs through the objective. The laser was coupled into the optical path with a dichroic mirror (Semrock) and the power in front of the objective was close to 900 mW with a pulse duration of 1.2 µs. A static magnetic field *B*_0_ between 30 and 40 mT was applied with a permanent magnet along the crystal axis of the subset of NVs used for NMR detection. The fluorescence of the other three directions was quenched by the off-axis field, but still contributed to the background. We always recorded two variants of a given measurement, one projecting the result onto the |0〉 spin state, the other to |1〉 (by a final MW pulse along +*σ*_*x*_ or −*σ*_*x*_, respectively) to remove common mode background by calculating the Michelson contrast (*I*_0_ − *I*_1_)/(*I*_0_ + *I*_1_) between the associated intensities. During one exposure of the camera, we typically recorded the light from 10,000 to 50,000 repetitions of a measurement sequence. We recorded several exposures for each combination of experimental parameters for additional averaging. The durations of a single exposure were 4 to 5 seconds in the standard NMR measurements. The 70 nm pattern became visible within about 20 minutes measurement time, but required additional averaging. The total raw measurement times after averaging for the combined ^1^H and ^19^F spectra resulting in the images in Figs 1 and 2 were about 9.6 hours and 11.7 hours, respectively. The raw measurement time for an exposure with Rabi readout was about 1 second (at reduced saturation of the camera), with a total of 11.3 hours. Additional experimental overhead could in principle be reduced close to zero, but with our current apparatus resulted in up to 24 hours total measurement time.

### Drift correction and upsampling

In the data set for the Rabi readout method, we used cross-correlation between the measurement frames to determine the drift in *xy*-direction of the optical microscope over the course of a measurement. Using the information about drift, we resampled and averaged the exposures taken with the same experimental parameters to obtain a single set of average frames on which the analysis was performed. According to the analysis, drift was typically only a fraction of a pixel. We upsampled the frames by a factor of two, to be able to correct drift at a fraction of a whole pixel. The analysis of the spatial resolution was based on the same data set in both cases, with and without spectral filtering, respectively. We hardly noticed drift in the *z*-direction along the optical axis. Additional details are given in the Supplement.

### Pulse engineering with GRAPE

We applied gradient ascent pulse engineering (GRAPE) to unitary propagators of the spin state with the aim to obtain robust *π* rotations irrespective of the initial state, using the average trace fidelity as the ensemble performance function^29^. In the secular approximation and rotating frame, the Hamiltonian for one NV in the ensemble is

$$ {\mathcal H} =D{S}_{z}^{2}-(\gamma {B}_{0}+{\rm{\Delta }}){S}_{z}-\beta \gamma ({B}_{x}(t){S}_{x}+{B}_{y}(t){S}_{y}).$$

The first two terms describe constant contributions, where *D* is the axial zero field splitting parameter of the NV, *B*_0_ is a constant field aligned along the NV axis and Δ is the sum of instantaneous sources of detuning from the applied MW frequency. Microwave control over the NV spin is expressed by the last term with time-dependent *x*- and *y*-components. The optimal control pulses are a pair of step-wise constant control amplitudes for these components of the MW field, equivalent to modulating its amplitude and phase. The GRAPE algorithm offers an elegant way to find local optima within this optimisation problem by providing a search direction. We built the ensemble around a central Rabi frequency of e.g. Ω_0_ = 20 MHz, which in itself would result in a rectangular *π* pulse of 25 ns duration on resonance. For the *π* pulse, we optimised for deviations of the Rabi frequency Ω from Ω_0_ in a range *β* = Ω/Ω_0_ = 0.5 … 1.5 in steps of 0.05. We aimed at as short pulses as possible, which necessarily kept the amplitude of the control fields high, such that possible detunings due to ^13^C hyperfine coupling were mostly covered and we included the hyperfine splittings due to both, ^14^N and ^15^N, i.e. Δ = 0, ±1.5 and ±2.2 MHz. Inhomogeneities of the static magnetic field *B*_0_ were on the order of 1 MHz across the whole field of view. We employed random but smooth control fields as starting guesses, limited in bandwidth to few tens of MHz, far below the measured 200 MHz modulation bandwidth of the hardware. The temporal resolution within the pulse was 1 ns. We then optimised pulses for a range of durations to find a duration for which a fidelity of ≥0.99 could be achieved. For example, the pulse used in Fig. 1 has a calculated fidelity of 0.991 at a duration of 122 ns. We aimed to apply the OC pulses at *B*_0_ ≈ 400G, and treated the NV as an effective spin 1/2 system in the calculation. Evaluating the fidelity of a finished pulse for spin 1 in the subspace spanned by |0〉 and |1〉 yielded no significant deviation from the spin 1/2 case. We optimised *π*/2 pulses for a broader range than the *π* pulses of *β* = 0.3 … 0.7, in order ensure that the *π* pulse limited the width of the decoupling and not the initialisation and final projection. We used the resulting *π*/2 pulse in all measurements (including those with rectangular *π* pulses) in order to unambiguously attribute the observed differences to the different *π* pulses. The *π*/2 pulse had a fidelity of 0.992 at a duration of 198 ns. Phase shifted *π*-pulses for XY16 (e.g. *π*_*y*_) where created by adding the appropriate phase to the *x*, *y* controls of the OC *π*_*x*_ pulse, since the static terms of ${\mathcal H}$ only included *S*_*z*_.

### Surface pattern

CaF_2_ was deposited on the diamond surface using electron-beam physical vapor deposition (EBPVD) through a transmission electron microscope grid. AFM measurements were obtained in a commercial system (Veeco). The observed steps at the transition to the CaF_2_ patches in NV and AFM measurements were well described by the convolution of a unit step with a normal distribution, $y=c+\frac{a}{2}{\rm{Erfc}}(\frac{x-{x}_{0}}{\sqrt{2}\sigma }),$ where Erfc is the complementary error function, *c* is a vertical offset, *a* is the height of the step, *x*_0_ is the centre of the transition and *σ* is the standard deviation of the normal distribution. We refer to *σ* as the step width within the main text.

### Reconstruction of NV depths

The model for the reconstruction of NV depths assumed a nuclear spin density at the surface composed of three or two stacked layers (with and without CaF_2_, respectively). The layers contributed (i) the proton signal typically seen in NMR with shallow NVs, attributed to adsorbed water, (ii) the fluorine nuclei in the residue of PTFE, and (iii) patterned fluorine nuclei where CaF_2_ had been deposited. The corresponding dataset contained average data including (i), (ii) and (iii) from both, 1.2 nm and 70 nm CaF_2_, respectively and (i) and (ii) outside of the patches, each of them for for *N* = 16, 32, 128, 160, and 256. In this way, a total of 15 data traces like those shown in Fig. 2f were obtained, each containing the ^19^F and ^1^H feature. ${B}_{{\rm{rms}}}^{2}$ is a sum over the contributions of the individual nuclei, which allowed us to separately model each of (i), (ii), and (iii) as a layer of finite thickness. Knowing the measured thicknesses of the CaF_2_ patches and the the bulk density of fluorine nuclei in CaF_2_ of about 4.9 × 10^28^ m^−3^, we left the spin densities and thicknesses of the proton and PTFE surface layers as free parameters. An additional depletion zone attributed for low stability of NVs within the first few nanometers from the surface, and was modelled as a transition of the NV yield from zero to one with a cos^2^ shape, leaving centre and width as free parameters. We combined the data in a least-squares fit to determine the free parameters, including the relative weights of the three CTRIM profiles. The following parameters were obtained: The relative weights of the 0°, 3°, and 7° profiles were 0.7(3), 0.3(3) and 0.0(3), respectively. The thickness fo the ^1^H surface layer was found to be 1.5(4) nm, with ${T}_{2{,}^{1}{\rm{H}}}^{\ast }=33(6){\rm{\mu }}s$ and a spin density of 6.1 × 10^28^ m^−3^. The thickness fo the ^19^F surface layer was found to be 4.0(2) nm, with ${T}_{2{,}^{19}{\rm{F}}}^{\ast }=18(2){\rm{\mu }}s$ and a spin density of 1.7 × 10^28^ 10^−3^. The value of ${T}_{2{,}^{19}{\rm{F}}}^{\ast }$ is on the order expected from dipolar coupling between next neighbor spins, while the higher value obtained for ${T}_{2{,}^{1}{\rm{H}}}^{\ast }$ might be due to motional narrowing e.g. in adsorbed water molecules. The depletion zone was found to extend to a depth of 4.0(1) nm and then rise to full yield within another 2.6(2) nm.

### Rabi oscillations and periodograms

The time domain data of the Rabi oscillations recorded in the Rabi readout method were well described by sinusoidal oscillations damped by stretched exponential decays, i.e. ∝ exp[−(*τ*_R_/*T*)^b^] × sin(Ω*τ*_R_ + *ϕ*_0_) with values of *T* around 75 to 200 ns, *b* about 0.4 to 0.5, and phase *ϕ*_0_ ≈ 0 (due to starting from a superposition state). Classical periodograms of the Rabi oscillations were obtained from the Fast Fourier Transform (FFT) of the Rabi oscillations, *P* ∝ |FFT|^2^. Band-limited interpolation between the raw data points was obtained by extending the recorded data points by a factor of 8 with zeros (zero-padding). We observed the shift of the interpolated periodograms to be consistent with the Rabi frequencies obtained from least-squares fits to the time-domain data (Supplementary Information).

## Supplementary information

Quantitative nanoscale MRI with a wide field of view (Supporting Information)

## Data Availability

Amplitude data of the optimal control pulses, and experimental data are available from the authors upon request.
