# Supplementary material for: Quantitative nanoscale MRI with a wide field of view
Source: Sci Rep. 2019 Aug 21;9:12166. doi: 10.1038/s41598-019-47084-w (PMC6704114; doi:10.1038/s41598-019-47084-w)
Supplement: Supplementary file 1 — Quantitative nanoscale MRI with a wide field of view (Supporting Information) [file 41598_2019_47084_MOESM1_ESM.pdf]

# Quantitative nanoscale MRI with a wide field of view (Supporting Information)

**F. Ziem[1,2,3,*], M. Garsi[1,2,3], H. Fedder[1,2,3,4], and J. Wrachtrup[1,2,3]**

[1]3rd Physical Institute, University of Stuttgart, Germany
[2]Institute for Quantum Science and Technology, IQst
[3]Center for Applied Quantum Technology
[4]Swabian Instruments GmbH, Frankenstr. 39, 71701 Schwieberdingen, Germany
[*]f.ziem@pi3.uni-stuttgart.de

## Contents

1 Rabi profile 2
2 Sensitivity with finite pulse durations 2
3 Scaling of pulse error with duration of the optimal control pulses 3
4 $B_0$ inhomogeneity 4
5 Integrals over decay data 4
6 Drift correction and upsampling 6
7 Periodograms of the Rabi oscillations 7
8 Simulation of Rabi Readout 7
References 8

## 1 Rabi profile

The Rabi frequencies of an NV are given by the component of the driving MW field perpendicular to the defect axis $\langle 111 \rangle$. If the amplitude of this component is $B_1$, the effective amplitude reduces to $B_1/2$ in the rotating wave approximation. In the following, we derive the Rabi frequencies within the field of the wire, using the following definitions: $I$ : peak current in the wire, $\hat{\boldsymbol{I}}$ : direction of the wire, $\boldsymbol{r}$ : position of the NV, $\boldsymbol{r}_\mathrm{w}$ : a reference point on the wire.

A point on the wire may be given by $\boldsymbol{r}' = \boldsymbol{r}_\mathrm{w} + s\hat{\boldsymbol{I}}$ and the minimal distance vector pointing from the closest point on the wire to the NV is

$$\boldsymbol{r}_\mathrm{min} = \boldsymbol{r} - \boldsymbol{r}_\mathrm{w} - s_\mathrm{min}\hat{\boldsymbol{I}} = \boldsymbol{r} - \boldsymbol{r}_\mathrm{w} + ((\boldsymbol{r}_\mathrm{w} - \boldsymbol{r}) \cdot \hat{\boldsymbol{I}})\hat{\boldsymbol{I}}.$$

The magnetic flux vector is then given by

$$\boldsymbol{B}(\boldsymbol{r}) = \frac{\mu_0 I}{2\pi|\boldsymbol{r}_\mathrm{min}|}\mathrm{Unit}\left[\hat{\boldsymbol{I}} \times \boldsymbol{r}_\mathrm{min}\right] \tag{1}$$

$$= \frac{\mu_0 I}{2\pi|\boldsymbol{r}_\mathrm{min}|}\left(\hat{\boldsymbol{I}} \times \frac{\boldsymbol{r}_\mathrm{min}}{|\boldsymbol{r}_\mathrm{min}|}\right) \tag{2}$$

where Unit[...] denotes taking the unit vector.

Within the geometry of the experiment (lab frame), we can assume the following vectors and directions:

$$\hat{\boldsymbol{I}} = (1,0,0)^\mathrm{T}, \tag{3}$$

$$\boldsymbol{r}_\mathrm{w} = (0,0,r_\mathrm{w})^\mathrm{T}, \tag{4}$$

$$\boldsymbol{r} = (0,y,0)^\mathrm{T}, \text{ and} \tag{5}$$

$$\hat{\boldsymbol{z}}_\mathrm{NV} = (1,0,1)^\mathrm{T}/\sqrt{2}, \tag{6}$$

where $\hat{\boldsymbol{z}}_\mathrm{NV}$ is the unit vector in direction of the NV axis. Using these definitions, we arrive at the following expressions for the parallel and perpendicular components of $\boldsymbol{B}$ at the position of a given NV:

$$B_\parallel(y) = \frac{\mu_0 I}{2\sqrt{2}\pi} \cdot \frac{y}{(r_\mathrm{w}^2 + y^2)}, \tag{7}$$

$$B_\perp(y) = \frac{\mu_0 I}{2\sqrt{2}\pi} \cdot \frac{\sqrt{2r_\mathrm{w}^2 + y^2}}{(r_\mathrm{w}^2 + y^2)}. \tag{8}$$

We can interpret $B_\perp$ as the result of the peak flux of an AC field created by the microwave currents. At the current frequencies and distances, retarded components of the field are of order $\mathscr{O}(10^{-3})$ and are neglected.

The $B_1$ term in the microwave Hamiltonian can be replaced by $B_\perp$, which results in the following expression for the Rabi frequency at distance $y$ from the wire:

$$\Omega(y) = \sqrt{\Omega_0^2(y) + \Delta^2} \tag{9}$$

$$= \sqrt{(\gamma B_\perp(y)/2)^2 + \Delta^2} \tag{10}$$

$$= \sqrt{\frac{\mu_0^2 I^2 \gamma^2}{32\pi^2} \cdot \frac{2 + (\frac{y}{r_\mathrm{w}})^2}{r_\mathrm{w}^2(1 + (\frac{y}{r_\mathrm{w}})^2)^2} + \Delta^2}, \tag{11}$$

where $\Delta$ is a detuning of the microwave from the NV spin transition. The Rabi profiles in our experiment were well described by this expression, when leaving $\Delta$ as a free parameter for an effective detuning. At low field strengths corresponding to Rabi frequencies $\leq 10\,\mathrm{MHz}$, further corrections due to the distribution of hyperfine coupling to $^{13}C$ nuclei may become necessary.

## 2 Sensitivity with finite pulse durations

The phase accumulated due to a time-dependent magnetic field during an interferometric sequence is given by

$$\Phi = -\int_0^\tau \gamma B_z(t) g(t)\, dt\,,$$

where $\gamma$ is the gyromagnetic ratio of the sensor, $B_z$ is the component of the field along the NV axis and $g$ is the time-domain sensitivity function generated by the decoupling pulses in the present case. Ideally, $g$ switches between $-1$ and $+1$ with every

switch of sign of $B_z$ to lead to a maximum accumulation of phase. In the frequency domain, $g$ can be expressed via its Fourier transform $G(f)$. This transformation is detailed in the following. We can decompose $g(t)$ into a few building blocks:

$$g = \Pi_T \times [(III_\tau^{\mathscr{O}} - III_\tau^{\mathscr{E}}) * \Pi_{\tau'}]. \tag{12}$$

Here, the periodicity of $g(t)$ is described using two Dirac combs $III_\tau^{\mathscr{O}}$ and $III_\tau^{\mathscr{E}}$, which we use to denote Dirac combs which only include terms at the odd and even multiples of $\tau$, respectively. The Dirac combs define the center of each sensing interval of duration $\tau'$, represented by the convolution $(*)$ with a unit step $\Pi_{\tau'}$. In this model, there is no phase accumulation during the pulse when $\tau' < \tau$. The overall duration of the sequence is obtained by multiplying $(\times)$with a unit step of duration $T$. Application of the Fourier transform to $g(t)$ results in

$$\begin{aligned} G(f) &= \mathscr{F}\{\Pi_T\} * [(\mathscr{F}\{III_\tau^{\mathscr{O}} - III_\tau^{\mathscr{E}}\}) \times \mathscr{F}\{\Pi_{\tau'}\}] \\ &= T\,\mathrm{sinc}(fT) * [(\mathscr{F}\{III_\tau^{\mathscr{O}}\} - \mathscr{F}\{III_\tau^{\mathscr{E}}\}) \times \tau'\mathrm{sinc}(f\tau')] \\ &= T\,\mathrm{sinc}(fT) * [\mathscr{F}\{III_\tau^{\mathscr{O}}\}(1 - e^{i2\pi f\tau}) \times \tau'\mathrm{sinc}(f\tau')] \\ &= T\,\mathrm{sinc}(fT) * \left[\frac{(1 - e^{i2\pi f\tau})}{2\tau} III_{(2\tau)^{-1}} \times \tau'\mathrm{sinc}(f\tau')\right], \end{aligned} \tag{13}$$

where the time shifting property of the Fourier transform was applied and the Dirac comb $III_\tau^{\mathscr{E}}$ which contained only even terms was replaced with the comb $III_{2\tau}$ of twice the period, but all terms. Next, the convolution can be evaluated:

$$G(f) = \sum_{k=-\infty}^{\infty} T\,\mathrm{sinc}\left(T(f - \frac{k}{2\tau})\right) \frac{(1 - e^{i\pi k})}{2\tau} \tau'\mathrm{sinc}\left(\frac{k\tau'}{2\tau}\right). \tag{14}$$

Due to the properties of the $\delta$-distribution, the convolution resulted in evaluating the terms at the frequencies $k/2\tau$ given by the Dirac comb $III_{(2\tau)^{-1}}$. Notably, all terms with even $k$ vanish and relabeling $k \mapsto 2k+1$ results in

$$G(f) = \sum_{k=-\infty}^{\infty} T\,\mathrm{sinc}\left(T(f - \frac{2k+1}{2\tau})\right) \frac{\tau'}{\tau}\mathrm{sinc}\left(\frac{(2k+1)\tau'}{2\tau}\right). \tag{15}$$

The sum now extends over the different harmonic orders of the filter, with $k \in \{-1, 0\}$ giving the main lobes in the frequency domain at $\pm(2\tau)^{-1}$.

The acquired phase can be expressed as $\Phi = -\int_0^\tau \gamma B_z(t)\mathscr{F}^{-1}\{G(f)\}\,\mathrm{d}t$, with the inverse Fourier transform $\mathscr{F}^{-1}$. The factors including $\tau'$ do not include $f$ and result in scaling factors to the phase due to the linearity of the Fourier transform. Hence, the relative change in acquired phase compared to the instantaneous $\pi$-pulse (for which $\tau' = \tau$), is given by the expression from the main text, $\kappa = \frac{\tau'}{\tau}(\mathrm{sinc}(\frac{\tau'}{2\tau})/\mathrm{sinc}(\frac{1}{2}))$. The relative contrast in phase noise spectroscopy is given by $\exp(-\langle\Phi^2\rangle/2)$, where $\langle\Phi^2\rangle \propto B_{\mathrm{rms}}^2$. The relative change in accumulated phase reflects on $B_{\mathrm{rms}}$ and in order to obtain the same relative contrast, $B_{\mathrm{rms}}$ needs to increase by a factor of $1/\kappa$. On the other hand, since $\exp(-\kappa^2 x) \approx 1 - \kappa^2 x$, a reduction of the acquired phase by $\kappa$ reduces the change in the signal for a given $B_{\mathrm{rms}}$ by $\kappa^2$. In the shot noise limit, this requires an increase of the measurement resources by $1/\kappa^4$.

## 3 Scaling of pulse error with duration of the optimal control pulses

For a given combination of detunings and Rabi inhomogeneities, we optimized $\pi$ rotations across a range of durations and with random starting guesses. In Fig. S1 we show the error (defined as 1 - fidelity) of the best optimal control pulses out of several different ensembles obtained via our GRAPE implementation. The ensembles used for optimization covered detunings due to the hyperfine coupling strengths of both, $^{14}$N and $^{15}$N in the NV, and a range of driving strength inhomogeneities $\beta = \Omega/20\,\mathrm{MHz}$ as indicated by the legend. The step size of $\beta$ was 0.05. The starting guesses for the controls were random, but the bandwidth was limited to 170 MHz throughout the optimization. The solid lines indicate the lowest error (pulses with best fidelity) obtained up to a given duration and indicate the lower boundary for the pulse error. For the blue lines, the lower value of the range of $\beta$ decreases until a value of 0.3 is reached. The larger end of the $\beta$-range increases steadily across the sets of pulses. Notably, the lower boundary of the error increases strongly until $\beta = 0.3$ is the smallest driving strength in the ensemble. The increase of the upper boundary of $\beta$ can be absorbed without major increases of the error. In other words, synthesis of the $\pi$ rotation is most expensive at low driving strength, presumably due to competition with the drift part of the Hamiltonian. In the pulses shown here, the error of the best results decays approximately exponentially with the duration of the pulse.

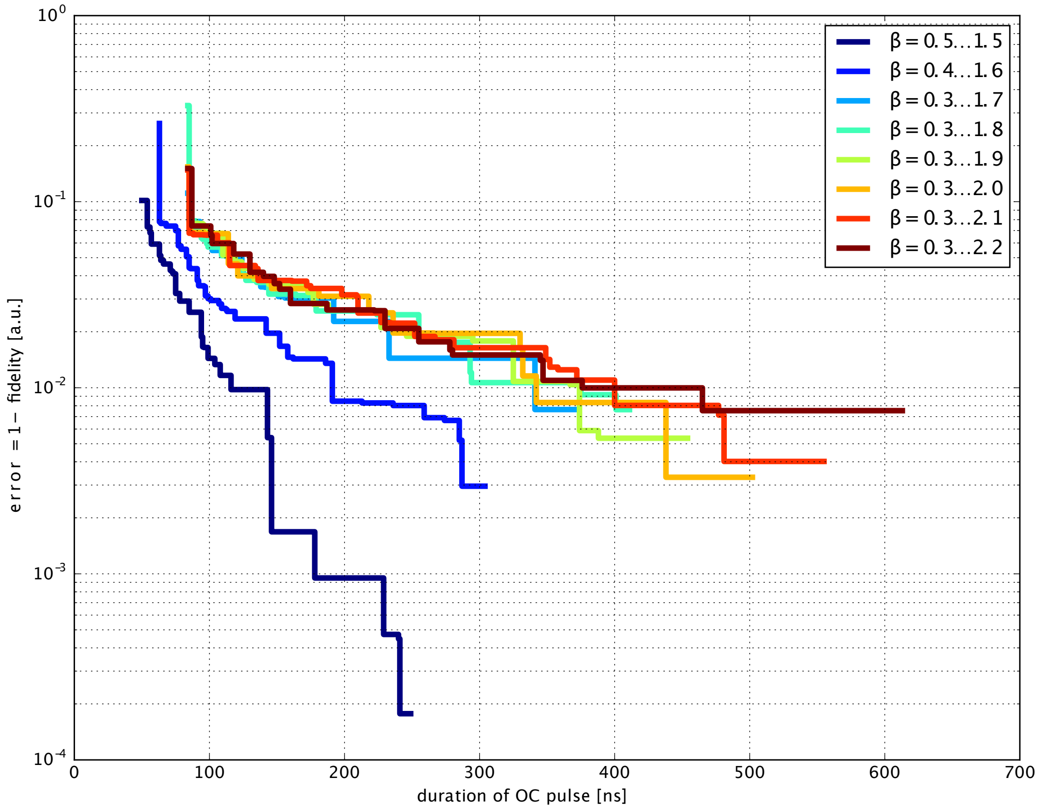


**Figure S1.** Scaling of the error of several sets of optimal control pulses. The solid lines give the lowest error obtained for a given duration out of a set of 2000 OC results. The pulses were optimized to cover ranges $\beta = \Omega/\Omega_0$, relative to $\Omega_0 = 20\,\mathrm{MHz}$.

## 4 $B_0$ inhomogeneity

Fig. S2 shows an example distribution of the fitted centers of the NV resonances lines across the field of view. For this measurement, $2\times 2$ pixels were binned on the camera, resulting in $256\times 256$ pixels. The data shown in this figure encompasses about 99.8% of all pixels. The remaining pixels were outliers, especially in the corners of the field of view, where the laser intensity was lower due to the illumination beam profile. Typically, fits with normal distributions to these histograms resulted in standard deviations of less than 2 MHz, corresponding to an inhomogeneity of the static magnetic field of about 70 µT across the field of view. The inhomogeneity in resonance frequencies was dominated by a remaining gradient of the static magnetic field across the field of view, such that the histogram of resonance frequencies need not be normal, but may change depending on the orientation of the static gradient. For the Rabi readout method, the gradients were extracted locally over a width of a few micrometers only, minimizing the effect of static detuning on the Rabi frequencies.

## 5 Integrals over decay data

In the following we motivate the use of the integrated $T_2$ decay data ($\Sigma$ in the main text) as a benchmark for the efficacy of dynamical decoupling. In the regime where dynamical decoupling fails, little signal-to-noise is left in the acquired data and the results extracted by least squares fitting routines become volatile. While robust estimators like the median data point along a decay enable qualitative comparison between well controlled and badly controlled subensembles, the integral could also be used to estimate $T_2$ with relatively little additional requirements. We start by noticing that by normalizing our data using the Michelson contrast, there is practically no offset baseline in the data, i.e. the decay is always towards zero. If that were not the case, a robust estimate of the baseline would be necessary in the following discussion. Assume now, that we are able to continuously record an exponential decay with amplitude $A$ and lifetime $T$ (e.g. $T_2$ or $T_1$), and with additional noise term $\delta$. For simplicity, we integrate our record from a starting time $t_0 \geq 0$ to infinity:

$$I = \int_{t_0}^{\infty} A\exp(-\tau/T) + \delta\,\mathrm{d}\tau = AT\exp(-t_0/T) + \int_{t_0}^{\infty} \delta\,\mathrm{d}\tau, \tag{16}$$

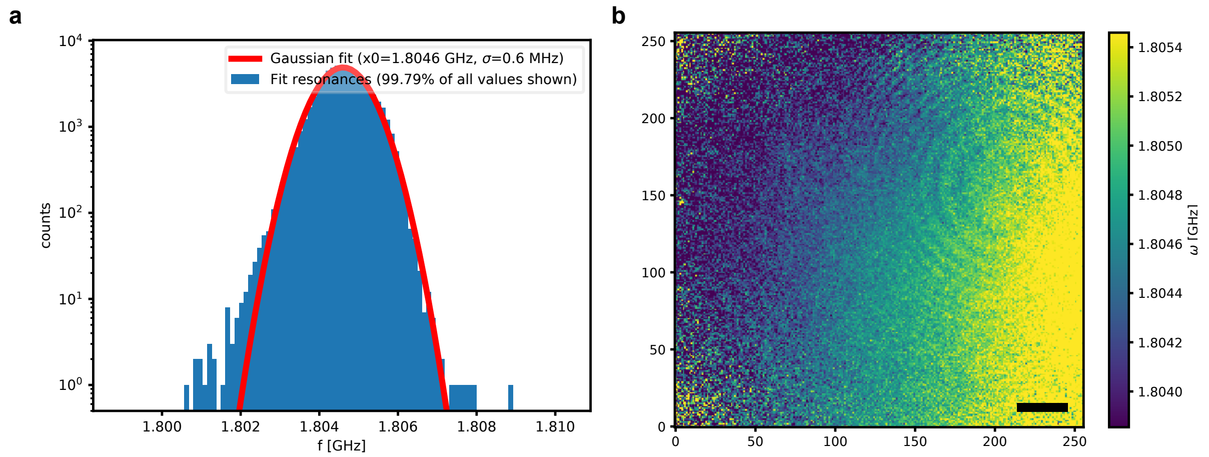


**Figure S2.** Example distribution of the NV resonance frequencies. **a** Histogram of fit resonance frequencies across the field of view. **b** Distribution of the resonance frequencies across the field of view with hardware binning to $256 \times 256$ pixels. Scale bar: $10\,\mu$m.

where the integral over the noise in the second term vanishes for identically distributed noise with short autocorrelation times. For a finite record up to a maximal value $t_1$, the noise needs to be reduced experimentally. If $t_0 > 0$, our record does not include the value of the amplitude $A$ and we replace it in eq. (16) with with an appropriately scaled amplitude $A = A_{\tau'} \exp(\tau'/T)$, with a supporting point $A'$ recorded at time $\tau' \geq t_0$, to obtain

$$I = A_{\tau'} T \exp((\tau' - t_0)/T). \tag{17}$$

In the case $\tau' = t_0$, we have $T = I/A(t_0)$ and can extract the lifetime of the decay from the integral over our record and the first recorded value at $t_0$.

In the main text, we used this expression and approximated the value of $I$ by a Riemann-type midpoint summation over the discrete data points. In the following, we add additional remarks on the applicability and alternate implementations of this scheme and lower boundaries set on $T$ extracted in this way.

For a choice of the supporting point $\tau' > t_0$, i.e. at a point in the record other than the initial data point, we can estimate the decay time using the general solution to eq. (17),

$$T = \frac{t_0 - \tau'}{W\left((t_o - \tau')A_{\tau'}/I\right)}, \tag{18}$$

where $W$ is the Lambert-W function. This expression is also valid when the integral (eq. 16) does not extent to infinity but to some finite value $t_1$, such that $t_0 < \tau' \leq t_1$, which is naturally the case for experimental data. The Lambert-W evaluates to real values only for real arguments $\geq -1/e$, such that generally certain combinations of recorded values may lead to unphysical results. Examples include errors of the recorded values and if the actual physical mechanism results in deviations from a monoexponential decay.

In the discrete case, we replace the integral in (eq. 16) with a sum over the set of $N$ data points $(\tau_k, y_k)$, $k = 1, \ldots, N$, to find:

$$\Sigma = \sum_{k=1}^{N} y_k \Delta_k = y_m \sum_{k=1}^{N} \exp((\tau_m - \tau_k)/T)\Delta_k, \tag{19}$$

where $\Delta_k$ is the 'bin size' occupied by data point $k$, i.e. the spacing between data points in case of equally spaced data. In this example, a Riemann-type midpoint summation is used as a robust approximation to the integral based on the measured data. To extract $T$ from this equation, an arbitrary data point $(\tau_m, y_m)$ can be chosen as the reference data point for the overall amplitude, but in practice one should choose $\tau_m \ll T$, such that $y_m$ is sufficiently larger than zero. The term $\tau_m/T$ in the exponent within the sum can then be neglected.

The relaxation time $T$ can in both cases, continuous and discrete, be evaluated from the integral over the recorded data and one reference data point, or a small subset of reference data points. The choice of these 'special' data points (at $\tau'$ and $\tau_m$,

respectively), or generally the estimate of the amplitude $A$, can have great influence on the overall result, especially due to noise. Since short evolution times also result in short measurements, a high number of repetitions can be chosen to improve the signal-to-noise ratio of the reference data. Due to the averaging effect of the integral or sum, higher noise levels can then be tolerated for the remaining data points.

The implicit and explicit simplifications and assumptions made above almost exclusively lead to an underestimation of $T$, such that the values extracted in this way represent a lower boundary. It should be noted, that any underestimation of the integral over data results in an underestimation of the $T$ value and that all of the following deviations from the ideal monoexponential model result in such a underestimation of the integral.

Mostly, a monoexponential decay does not describe the shape of the experimentally observed decay curves well, and usually a stretched exponential decay, $\exp(-(\tau/T)^b)$, is applied. If we evaluate the integral over the stretched exponential form, we obtain

$$I_b = \int_0^\infty \exp(-(\tau/T)^b)\,\mathrm{d}\tau = AT\,\Gamma(1+1/b).$$

The Gamma function in this expression introduces an additional factor $\Gamma \approx 1$ for the relevant range of $0.5 < b < 2$. In the case of dense ensembles of shallow NVs, we have so far only observed $b < 1$, for which $I_b \gtrsim I$. Hence, neglecting $b$ leads us to underestimate the value of $T_2$.

The situation changes if we start our integration at a finite time $t_0$, such that the altered shape of the decay compared to a monoexponential decay affects the integral over the data:

$$I_{b,\mathrm{inc}} = \int_{t_0}^\infty \exp(-(\tau/T)^b)\,\mathrm{d}\tau = \frac{AT}{b}\Gamma(1/b,(t_0/T)^b),$$

where $\Gamma(a,z) = \int_z^\infty t^{a-1}e^{-t}\mathrm{d}t$ is the incomplete Gamma function. For $t_0 \ll T$, the additional factor due to $\Gamma/b$ for $b \neq 1$ may drop to 0.9 around $b \approx 0.7$ and further reduces as $t_0/T$ increases. By including an appropriate correction factor into the analysis, overestimation of $T$ can be prevented. Alternatively, $b$ can also be estimated by using several reference points.

Additional modulations of the signal, like the dips observed due to the $^{13}$C spin bath, result in a reduction of the integral or sum over the signal. This also applies to the maximal $\tau$ used in measurements, which is finite and truncates the integral. The error in estimating $T$ in the case of truncation scales like the integral in equation 16, i.e. it decays exponentially. In the case of a stretched exponential decay with $b \leq 1$, the Riemann summation underestimates the integral. Finally, the number and choice of measurement times $\tau_k$ influences the accuracy of the estimate. We typically use quadratically increasing free evolution times, such that there is a higher density of data points where the slope of the decay is highest. This improves accuracy of the result compared to using the same number of equally spaced data points, while also reducing the measurement time. The error of the sum due to noise decreases as $\sqrt{N}$.

## 6 Drift correction and upsampling

The relative translation $\boldsymbol{u} = (u_x, u_y)$ between the frames recorded during the measurements was determined by pairwise cross-correlation. Taking a set of $N$ frames, there are $\binom{N}{2}$ possible pairs of frames, where $\binom{N}{k} = \frac{N!}{k!(N-k)!}$. In total, there are $2\binom{N}{2}$ equations for the $2N$ coordinates $x_i, y_i$:

$$\boldsymbol{u}_{ij} = \boldsymbol{r}_j - \boldsymbol{r}_i,$$

where $\boldsymbol{r}_i = (x_i, y_i)$ are the absolute positions of the frames within a common coordinate system and $\boldsymbol{u}_{ij}$ is the shift vector determined from the cross correlation. Admitting for noise in the counts at each pixel, the vectors $\boldsymbol{u}_{ij}$ will carry an error. $\binom{N}{k}$ is weakly lower bounded by $(N/k)^k$, such that the number of equations scales more strongly than $N^2$ for $k = 2$. The number of coordinates only increases to $2N$, such that the system of equations gets overdetermined and a least-squares solution to the equation $\boldsymbol{A} \cdot \boldsymbol{x} = \boldsymbol{b}$, made up from the relative positions determined by pairwise cross-correlation, improves the estimated coordinates (unless there are significant systematic errors in the $\boldsymbol{u}_{ij}$).

To keep the computational problem within reasonable bounds, we did not obtain the cross-correlation for all frames of a measurement, but only a subset of 16 frames which served as 'ground truth' reference to which all other frames were aligned. The reference frames were evenly spaced along the whole duration of the measurement and were taken from different combinations of the experimental parameters (interpulse distance $\tau$ used for locking onto the NMR signal, duration $\tau_\mathrm{R}$ of final Rabi drive, and direction of axis $+\sigma_x$ or $-\sigma_x$ along which the Rabi drive was applied). This diverse set of conditions was chosen to mitigate systematic effects in the cross-correlation which might have been caused by common patterns in NV brightness for similar parameters. We determined the pairwise cross-correlations between this set of frames at a resolution of 1/20th of a pixel, or about 8 nm, using the `skimage.feature.register_translation` function of the scikit-image Python

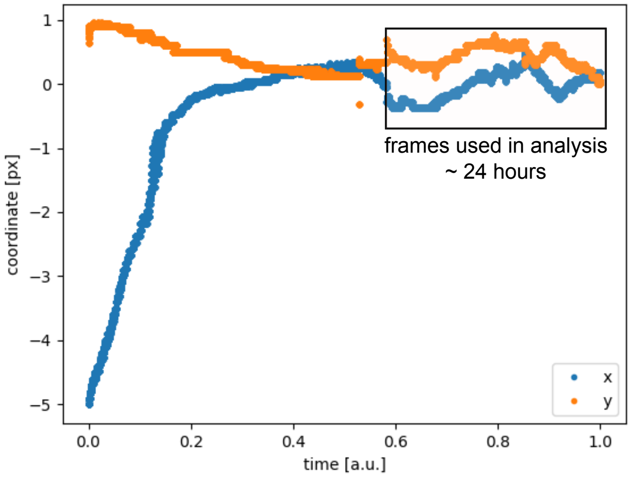


**Figure S3. Drift** The drift, measured in pixels, of the optical microscope determined from cross-correlation of the measurement frames with a set of reference frames. Only the set of frames obtained after a stabilization phase were taken for analysis of the Rabi readout methods explained in the main text.

library[1] and obtained the least-squares solution for the relative positions of the reference frames. Afterwards, we determined the relative shift of each frame from the measurement against each of the reference frames and took the average of the 16 positions obtained for a given frame to determine its position within the coordinate system established by the reference frames. Finally, we resampled the frames using cubic splines, upsampling by a factor of two during the process, i.e. doubling the number of pixels along each dimension $x$ and $y$ of the frames. Fig. S3 shows the drift of the optical microscope as determined by our analysis. The data used in analysis of the Rabi readout were obtained during a day of total measurement time (marked in the plot) during which we determined only sub-pixel drift which was corrected for during resampling.

## 7 Periodograms of the Rabi oscillations

Periodograms are estimators for the power spectrum of a signal. Fig. 3c in the main text shows classical periodograms of the Rabi oscillations obtained from Fast Fourier Transforms (FFT), which were interpolated between the raw data points by zero-padding the time-domain signal. The classical periodogram is

$$P(f) \propto |\sum_{n=1}^{N} g_n e^{-2\pi i f t_n}|, \tag{20}$$

where $N$ is the number of data points $g_n$ sampled over a total duration $T$ at evenly spaced times $t_n = T/N$. The FFT captures the complete frequency information when evaluated at frequencies $f_k = n/T$, with $n = -N/2$ to $N/2$. Zero-padding the data does not improve spectral resolution, it merely adds intermediate frequencies at which eq. (20) is evaluated. These interpolated spectra were also the basis for the Rabi readout, i.e. integration over small bands within these spectra correspond to the 'filtered' data points in Fig. 3d. To show that the interpolated spectra behave well around their maxima, we fit the peaks of the interpolated spectra with squared normal distributions to extract the dominant frequency contribution in a line of pixels perpendicular to the gradient within the Rabi frequencies. We compared the frequencies obtained in this way to the Rabi frequencies obtained from least-squares fits to the time-domain data. As shown in Fig. S4, the shift of the frequencies was nearly identical for both procedures, corresponding to a consistent extraction of the gradient in both domains. We observed a small constant offset between the absolut values, which was smaller than the width of the band used for band-filtering the spectra. In addition, the remaining relative fluctuations between the frequency values was about 1/10th of the width of the band used for filtering the spectra. We took the absolute values from the frequency-domain fits as the centers of the band-filtering to obtain the contribution of NV centers at each position along the gradient in the Rabi readout method.

## 8 Simulation of Rabi Readout

We performed numerical simulations of the Rabi readout for a range of gradients and intrinsic line widths using experimental conditions, including a signal-to-noise ratio of 5. Here we show an example for $|\nabla\Omega| = 5\,\mathrm{MHz}/\mu\mathrm{m}$. In this example, we

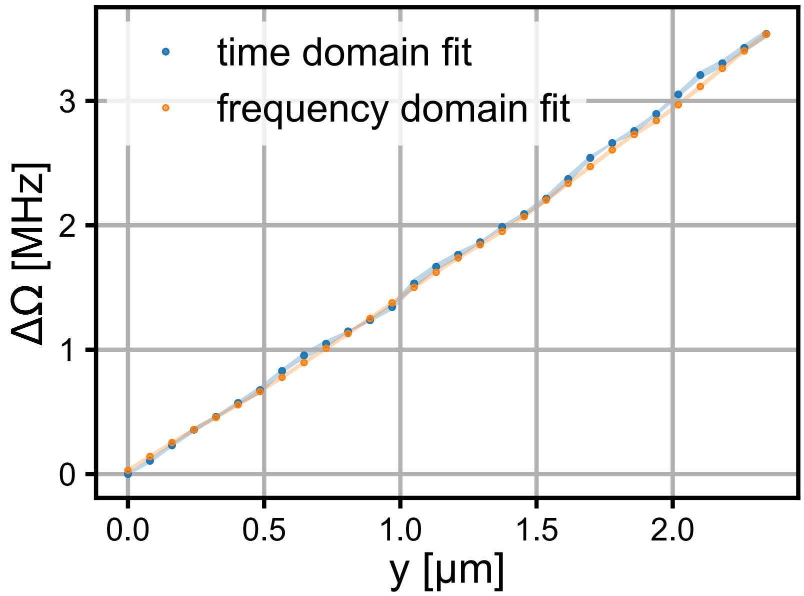


**Figure S4. Comparison of time-domain and frequency-domain fits** Shift $\Delta\Omega$ of the Rabi frequency obtained from least-squares fits in the time domain and in the frequency domain. Shaded areas correspond to the standard deviation of the fitted value. A small constant offset of 0.12 MHz has been added to the frequency domain data to simplify comparison between the relative differences.

simulated pixels of 80 nm in width, which may be obtained experimentally from supersampling during correction of frame drift. We generated a random distribution of NV coordinates and calculated the photon distribution due to the NVs with a Gaussian point spread function with standard deviation $\sigma_{\mathrm{opt}} = 165\,\mathrm{nm}$. We modeled the NMR signal as a Gaussian dip in the NV response around 1.5 MHz. The NMR signal was only applied at the lower half of the simulated frames. We applied the analysis of the Rabi spectra as described in the main text and obtained a significant reduction in the observed step width to 40 nm compared to the expected result of $\sigma_{\mathrm{opt}}$. Even without filtering, a slight improvement in step width to 144 nm was observed, which we attribute to a filtering effect of least-squares fitting to the data. The results of the simulation are shown in Fig. S5.

## References

**1.** van der Walt, S. *et al.* scikit-image: image processing in python. *PeerJ* **2**, e453, DOI: 10.7717/peerj.453 (2014).

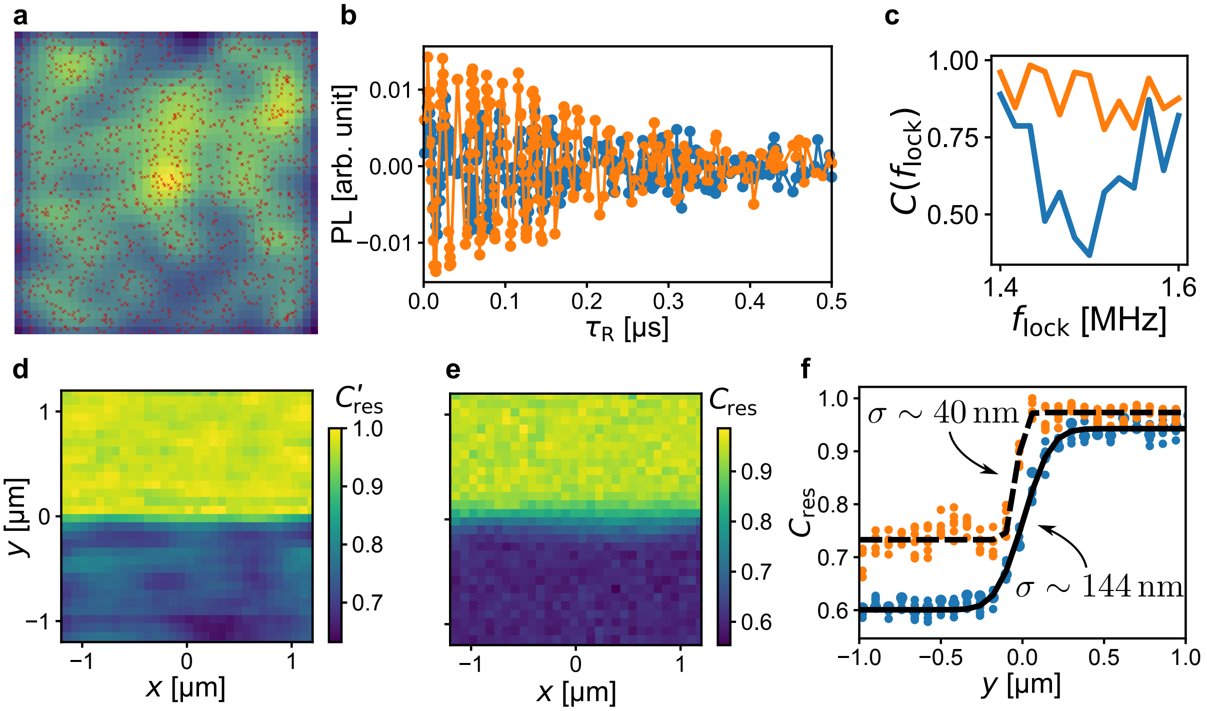


**Figure S5.** **Simulation of Rabi readout** In the simulation, an NMR signal was only applied at the lower half of the frame, with a sharp edge at $y = 0$. **a**, Diffraction limited image of randomly distributed NVs (red crosses). **b** Rabi osciilations modulated by NMR signal from a single pixel (orange: off resonance, blue: on resonance). **c** Signal strength of the traces in b, where $\tau_R$ corresponds to a $\pi/2$-pulse. Resonance was at $f_{\mathrm{lock}} = 1.5\,\mathrm{MHz}$. **d** MRI using Rabi readout. The NMR signal at the lower half of the frame results in reduced contrast. A sharp edge is observed. **e** MRI using the unfiltered signal. A smoother step is observed. **f** Comparison of step profiles with (orange) and without (blue) Rabi readout. The step width reduces to 40 nm with Rabi readout, compared to 144 nm without.
